# Association between parental socioeconomic status with underweight and obesity in children from two Spanish birth cohorts: a changing relationship

**DOI:** 10.1186/s12889-015-2569-5

**Published:** 2015-12-22

**Authors:** Vicente Martínez-Vizcaíno, Montserrat Solera-Martínez, Iván Cavero-Redondo, Jorge Cañete García-Prieto, Natalia Arias-Palencia, Blanca Notario-Pacheco, Maria Martínez-Andrés, Jorge Mota, Mairena Sánchez-López, on behalf of Cuenca Study Group

**Affiliations:** Universidad de Castilla-La Mancha: Health and Social Research Center, Cuenca, Castilla-La Mancha España; Facultad de Ciencias de la Salud, Universidad Autónoma de Chile, Talca, Chile; Research Centre in Physical Activity, Health and Leisure, University of Porto, Porto, Portugal; Universidad de Castilla-La Mancha: School of Education, Ciudad Real, Castilla-La Mancha España

**Keywords:** Child health, Weight status, Socioeconomic status

## Abstract

**Background:**

Our objective was twofold: to estimate the prevalence of underweight, overweight, and obesity in two birth cohorts (1999–2000 and 2007–2008) from Castilla-La Mancha, Spain; and to examine the association between parental socioeconomic status (SES) and weight status in these two cohorts.

**Methods:**

Cross-sectional analysis of baseline measurements was utilised in two cluster randomised trials. Using population-based samples of children from Castilla-La Mancha, Spain, 1158 children with a mean age of 9.5 years, born in the years 1999–2000 and 1588 children with a mean age of 5.3 years born in the years 2007–2008 participated. Children were classified according to the body mass index cut-offs proposed by the International Obesity Task Force criteria. An index of SES was calculated using questions regarding parental education and occupation levels.

**Results:**

Prevalence of underweight was higher in the 2007–2008 birth cohort (20.5 %, 95 % CI: 18.5, 22.5) than in the 1999–2000 birth cohort (8.1 %, 95 % CI: 6.5, 9.7), and the overweight/obesity prevalence was 20.4 % (95 % CI: 18.4, 22.5) and 35.5 % (95 % CI: 32.7, 38.3) respectively. In the lower SES stratum, in the 2007–2008 birth cohort, the prevalence of underweight and overweight/obesity was 36.7 % (95 % CI: 22.2, 51.2) and 16.3 % (95 % CI: 4.9, 27.7) respectively, and 22.2 % (95 % CI: 2.8, 60.0) and 55.5 % (95 % CI: 21.2, 86.3) in the 1999–2000 cohort. The ratio between underweight:overweight/obesity showed higher values for all SES categories in 2007–2008 cohort, but particularly in the lower SES group (0.4 in the 1999–2000 cohort and 2.2 in the 2007–2008 cohort).

**Conclusion:**

Underweight prevalence was lower in the cohort of children born in 1999–2000, and the prevalence of overweight and obesity was lower in the cohort of children born in 2007–2008. Furthermore, while in the 1999–2000 children’s cohort underweight was more frequent amongst children from high SES families and overweight/obesity was more frequent in children from low SES families, in the 2008–2009 children’s cohort the opposite was true.

**Electronic supplementary material:**

The online version of this article (doi:10.1186/s12889-015-2569-5) contains supplementary material, which is available to authorized users.

## Background

Prevalence of obesity and overweight have risen substantially in the past three decades in Spain [1] and most countries, even though marked variations in the estimations across countries have been reported [2]. The excess weight prevalence has grown steadily over the past two decades in Spanish children and also in children from most countries [3] even at early ages [4]. Paradoxically although excess weight in children is increasing worldwide, underweight remains a serious health problem in low income countries [5]. In Spanish children, the increasing trend in the prevalence of obesity has been accompanied by a similar trend in underweight prevalence [1].

The influence of several factors, including genetic, environmental, cultural, and socioeconomic status (SES) on weight status in children has been extensively described [6]. Childhood obesity and overweight have stabilised in England during the last decade, but children from lower SES have not benefited from this trend [7]. While in low income countries obesity in children has been associated with high parental SES and underweight with lower SES, in wealthy countries the opposite is true [8]. In Denmark an increased prevalence of overweight in children from low educational levels families has been attributed to social inequalities, thus it has been proposed that public health initiatives aimed at preventing and reducing excess weight should consider parents’ SES level [9].

Children’s obesity and parental socioeconomic position have been consistently related. Energy-dense diets are associated with lower daily food consumption costs and may be an effective way to save money. In addition low SES has been associated with less participation in sports or physical activities. However, it is unclear whether parental socioeconomic circumstances affect children’s weight status at a young age [10].

From 2008 until today the financial crisis has caused deterioration in the health of Europeans [11] and might have deteriorated the health and welfare of families from some European countries such as Greece, Spain, or Portugal. The economic downturn can be expected to reduce nutrition quality and physical activity, worsening obesity prevalence when society is least able to bear the escalating financial burden [12]. In some regions of Spain between 2005 and 2010, the proportion of children at risk of poverty increased from 20.6 % to 23.7 %, and living in unemployed families from 3.7 % to 11.2 % [11].

Our objective was twofold: first, to estimate the prevalence of underweight, overweight, and obesity in both the 1999–2000 and the 2007–2008 birth cohorts from Castilla-La Mancha, Spain; and second, to examine the association between parental socioeconomic levels and weight status in both cohorts of children.

## Methods

### Study design and population

#### Design

Cross-sectional analysis of the baseline measurements data from two cluster randomised trials aimed to evaluate the effectiveness of leisure-time physical activity on the prevention of childhood obesity: MOVI-2 study and Movi-KIDS study. Both studies included population-based samples of children from the Castilla-La Mancha region, Spain. Castilla-La Mancha is a central region of Spain, and has more than two million inhabitants (11.00 % immigrants from Eastern Europe, North-Africa, Asian, and South and Central-America) predominantly of low-medium socioeconomic level. The unemployment rate in the Castilla-La Mancha region has increased from approximately 12 % in 2008 to 30 % in 2013.

Participants belonged to two population-based birth cohorts:

- Year 1999–2000 cohort. Children born in the years 1999–2000 were recruited from the baseline measurements (September–November 2010) of a cluster randomised trial aimed to evaluate the effectiveness of a leisure-time programme of physical activity for the prevention of childhood obesity (clinicaltrials.gov number: NCT01277224). All the fourth and fifth grade primary school children (*n* = 1,592) belonging to 20 public schools from 20 towns in the Castilla-La Mancha province of Cuenca, Spain, were invited to participate, and 1,158 (72.7 % participation rate) accepted; of them, 544 completed questionnaires on parental SES [13].
- Year 2007–2008 cohort. Children born in the years 2007–2008 participating in the baseline measurements (September–November 2013) of an ongoing cross-over cluster randomised trial (clinicaltrials.gov number: NCT01971840) aimed also at promoting physical activity, but in children aged 4 to 7 years. Children of both third grade of pre-primary education and first grade of primary education (*n* = 2,407) belonging to 21 public schools from the Castilla-La Mancha provinces of Cuenca and Ciudad Real were invited to participate. Out of these 1,588 (66.0 % participation rate) accepted, and 1,398 completed questionnaires on parental SES [14].

### Ethical and legal aspects

The Clinical Research Ethics Committee of the Virgen de la Luz Hospital in Cuenca approved both cluster randomised trials. They were also approved by the Director and Board of Governors (Consejo Escolar) of each school. For data collection, parents gave written consent for their child to participate in the study, and children gave verbal consent when they were asked to collaborate in informative talks held class-by-class. After the data were gathered, the parents were informed by letter of their children’s results.

### Study variables

#### Anthropometry

In both the 1999–2000 and the 2007–2008 studies, anthropometric measurements were obtained at the schools by trained and certified nurses. *Weight* was calculated as the mean of two readings on a digital scale (100 g accuracy) with children lightly dressed and without shoes (Seca® 861, Vogel & Halke, Hamburg, Germany). *Height* was assessed as the mean of two measures with a wall-mounted height rod (Seca® 222, Vogel & Halke, Hamburg, Germany) on shoeless children standing straight against the wall, so that their spine was vertically aligned with the centre of the height rod. The head was placed with the chin parallel to the floor, and height was measured to the nearest mm. *Body mass index (BMI)* was calculated as weight in kg divided by the square of the height in metres. *Body fat percentage (BF%)* was estimated with electronic bio-impedance monitoring with the Tanita® BC-418 MA model of 8-contact electrode system (Tanita Corp. Tokyo, Japan) in MOVI-2 study and with the Tanita® Segmental-418 model of 4-contact electrode system (Tanita Corp. Tokyo, Japan) in Movi-KIDS study [15]. The body fat percentage was calculated as the mean of two readings obtained in the morning, under controlled temperature and humidity conditions, with the child being shoeless, fasting, after urination and a 15-minute rest, in the two cluster randomised trials.

#### Parental socioeconomic status

Data for parental SES were gathered by using self-reported occupation and education questions completed by either the father or the mother. Paternal and maternal education were classified separately as primary education (functionally illiterate, without any studies or had not completed primary education), middle education (primary education or high school/secondary education) and university education (university degree or PhD). Parental occupation was classified into five categories: 1. supervisor/manager or freelance with 10 employees or more, 2. supervisor/manager or freelance with less than 10 employees, 3. freelance with no staff, 4. non-qualified staff and unskilled worker, and 5. household chores, unemployed and others. An index of SES was calculated with the items that referred to the parents’ education and parental occupation classifying SES as: lower, lower middle, middle, upper middle, and upper according to the scale proposed by the Spanish Society of Epidemiology [16].

### Statistical analysis

Children were classified as underweight, normal weight, overweight, and obese according to the BMI cut-offs proposed by the International Obesity Task Force (IOTF) [17]. Differences in the frequency of each weight status category between boys and girls were assessed with the chi-squared test. Differences in body composition variables were tested by unpaired t-tests.

Differences in the mean of body composition variables by parental SES categories (five and three categories) were assessed using analysis of covariance (ANCOVA) controlling for age.

Logistic regression models were used to examine the association between three SES levels (middle SES was used as a reference category) and weight status categories. Odds ratios (ORs) and the 95 % confidence intervals (CIs) were estimated.

Analyses were performed with IBM SPSS Statistics 22 (SPSS, Inc., Chicago, IL), and statistical significance was set at two tailed p ≤0.05.

## Results

The mean age range at the examination in the 1999–2000 cohort was 8–11 years and 4–7 years in the 2007–2008 cohort. Using IOTF cut-off points, the prevalence of underweight was higher in the 2007–2008 birth cohort (20.5 %, 95 % CI: 18.5, 22.5) than in the 1999–2000 birth cohort (8.1 %, 95 % CI: 6.5, 9.7). Moreover excess weight prevalence (overweight/obesity) estimates were 35.5 % (95 % CI: 32.7, 38.3) for the 1999–2000 cohort and 20.4 % (95 % CI: 18.4, 22.5) for the 2007–2008 cohort. In both cohorts were similar the age, sex and anthropometric characteristics of those children whose parents responded to the SES questionnaire and those who did not do so (Table 1).

**Table 1:** Body composition variables, weight status and parental socio-economic status in the study cohorts, by familial response behavior

	Birth cohort					
	1999-2000 (*n* = 1158)			2007-2008 (*n* = 1588)		
	Respondents SES	Non-respondents SES	Total	Respondents SES	Non-respondents SES	Total
Boys, n (%)	253 (46.5)	334 (54.2)	587 (50.7)	715 (51.1)	91 (47.9)	806 (50.8)
Girls, n (%)	291 (53.5)	280 (45.5)	571 (49.3)	571 (48.9)	99 (52.1)	782 (49.2)
Age mean, years (range)	9.4 (8–11)	9.7 (8–11)	9.5 (8–11)	5.3 (4–7)	5.4 (4–7)	5.3 (4–7)
Body composition variables, mean (SD)						
Weight (kg)	37.3 (9.3)	37.4 (9.1)	37.3 (9.2)	21.3 (4.7)	21.7 (5.0)	21.4 (4.8)
Height (cm)	139.6 (7.2)	139.5 (6.8)	139.6 (7.0)	115.4 (6.1)	115.7 (5.8)	115.5 (6.1)
BMI (kg/m^2^)	19.0 (3.7)	19.0 (3.7)	19.0 (3.7)	15.9 (2.4)	16.0 (2.6)	15.9 (2.5)
BF%	25.4 (6.6)	25.2 (6.9)	25.3 (6.8)	20.1 (5.8)	20.5 (6.2)	20.2 (5.8)
Weight status categories, % (95 % CI)						
Underweight	7.5 (5.2-9.8)	8.6 (6.3-10.9)	8.1 (6.5-9.7)	20.5 (18.4-22.7)	20.5 (14.5-26.5)	20.5 (18.5-22.5)
Normal weight	57.4 (53.1-61.6)	55.4 (51.5-59.5)	56.4 (53.5-59.3)	59.2 (56.5-61.8)	57.9 (50.6-65.2)	59.0 (56.5-61.4)
Overweight	25.9 (22.1-29.7)	25.0 (21.6-28.6)	25.5 (22.9-28.0)	12.0 (10.3-13.7)	10.5 (5.9-15.1)	11.8 (10.2-13.4)
Obesity	9.2 (6.7-11.7)	10.7 (8.2-13.3)	10.0 (8.2-11.8)	8.3 (6.8-9.8)	11.1 (6.3-15.8)	8.6 (7.2-10.0)
Overweight/obesity	35.1 (31.0-39.2)	35.7 (31.9-39.7)	35.5 (32.7-38.3)	20.3 (18.2-22.5)	21.6 (15.5-27.7)	20.4 (18.4-22.5)
Parental socio-economic status, % (95 % CI)						
	*N* = 544			*N* = 1398		
Lower	1.6 (0.5-2.9)			3.5 (2.5-4.5)		
Lower middle	18.9 (15.5-22.3)			27.3 (24.9-29.7)		
Middle	38.2 (34.1-42.4)			44.8 (42.2-47.5)		
Upper middle	31.8 (27.8-35.8)			19.9 (17.8-22.1)		
Upper	9.4 (6.8-11.9)			4.4 (3.2-5.5)		

Abbreviations: *BMI* body mass index, *BF%* body fat percentage, *SD* Standard deviation, *CI* confidence interval

There were statistically significant gender differences in the mean of BF% (higher in girls, *p* < 0.001) and for BMI for age z-scores (higher in boys, *p* < 0.01) in the 1999–2000 cohort (Table 2).

**Table 2:** Gender differences in body composition and weight status categories, by birth cohort

	Birth cohort					
	1999-2000 (*n* = 1158)			2007-2008 (*n* = 1588)		
	Boys	Girls		Boys	Girls	
	*n* = 587	*n* = 571	p	*n* = 806	*n* = 782	p
Body composition variables, mean (SD)						
BMI (kg/m^2^)	19.2 (3.8)	18.8 (3.5)	0.1	16.0 (2.5)	15.8 (2.5)	0.2
BF%	23.9 (7.1)	26.8 (6.1)	**<0.001**	20.0 (5.2)	20.4 (6.4)	0.2
BMI-for-age z-score	0.9 (1.4)	0.7 (1.3)	**0.002**	0.2 (1.4)	0.1 (1.4)	0.1
Weight status categories, % (95 % CI)						
Underweight	8.5 (6.2-10.9)	7.7 (5.4-10.0)	0.69	19.9 (17.0-22.7)	21.2 (18.3-24.1)	0.54
Normal weight	53.8 (49.7-57.9)	59.0 (54.9-63.1)	0.08	61.4 (58.0-64.8)	56.5 (52.9-60.1)	0.05
Overweight	27.1 (23.4-30.8)	23.8 (20.2-27.4)	0.23	10.3 (8.1-12.4)	13.4 (11.0-15.9)	0.06
Obesity	10.6 (7.9-13.1)	9.5 (7.0-11.9)	0.60	8.4 (6.4-10.4)	8.8 (6.8-10.9)	0.85
Overweight/ obesity	37.7 (33.6-41.6)	33.3 (29.3-37.2)	0.13	18.7 (16.0-21.3)	22.2 (19.3-25.2)	0.09

Abbreviations: *BMI* body mass index, *BF%* body fat percentage, *CI* confidence interval, *SD* Standard deviation; in bold when *p* < 0.05

In the lower SES stratum, in the 2007–2008 birth cohort, the prevalence of underweight was 36.7 % (95 % CI: 22.2, 51.2), and 22.2 % (95 % CI: 2.8, 60.0) in the 1999–2000 cohort (Table 3). No remarkable differences were found in excess weight prevalence by parental SES between both cohorts. The ratio underweight: overweight/obesity showed higher values for all SES categories in 2007–2008 cohort, but particularly in the lower SES group (0.4 in the 1999–2000 cohort and 2.2 in the 2007–2008 cohort). Additional file 1 displays the frequency of weight status categories by parental SES with three categories in the 1999–2000 and 2007–2008 birth cohorts.

**Table 3:** Differences in frequencies of children in weight status categories by parental socio-economic status, controlling for age in the study birth cohorts

Birth cohort 1999–2000 (*n* = 544)						Birth cohort 2007–2008 (*n* = 1398)				
Parental socio-economic status										
	Lower	Lower middle	Middle	Upper middle	Upper	Lower	Lower middle	Middle	Upper middle	Upper
	*n* = 9	*n* = 103	*n* = 208	*n* = 173	*n* = 51	*n* = 49	*n* = 382	*n* = 627	*n* = 279	*n* = 61
Underweight	22.2 (2.8-60.0)	5.8 (0.8-10.8)	7.7 (3.8-11.5)	6.9 (2.9-11.0)	9.8 (3.3-21.4)	36.7 (22.2-51.2)	19.6 (15.5-23.7)	21.0 (17.8-24.3)	19.7 (14.9-24.6)	11.5 (2.6-20.3)
Normal weight	22.2 (2.8-60.0)	54.4 (44.3-64.5)	59.1 (52.2-66.0)	59.0 (51.3-66.6)	56.8 (42.2-71.4)	46.9 (31.9-61.9)	58.1 (53.0-63.2)	59.0 (55.1-62.9)	62.4 (56.5-68.2)	62.3 (49.3-75.3)
Overweight	33.3 (7.5-70.1)	25.2 (16.4-34.1)	25.5 (19.3-31.6)	28.3 (21.3-35.3)	19.6 (7.7-31.5)	6.1 (1.3-16.9)	13.1 (9.6-16.6)	11.3 (8.8-13.9)	11.8 (7.8-15.8)	18.0 (7.6-28.5)
Obesity	22.2 (2.8-60.0)	14.6 (7.3-21.9)	7.7 (3.8-11.5)	5.8 (2.0-9.5)	13.7 (3.3-24.1)	10.2 (3.4-22.2)	9.2 (6.1-12.2)	8.6 (6.3-10.9)	6.1 (3.1-9.1)	8.2 (2.7-18.1)
Overweight/ obesity	55.5 (21.2-86.3)	39.8 (29.9-49.7)	33.2 (26.5-39.8)	34.1 (26.7-41.4)	33.3 (19.4-47.2)	16.3 (4.9-27.7)	22.2 (17.9-26.5)	19.9 (16.7-23.1)	17.9 (13.2-22.6)	26.2 (14.4-38.1)
Ratio UW:OV/OB	0.4	0.1	0.2	0.2	0.3	2.2	0.9	1.0	1.1	0.4

Abbreviations: *UW* : OV/OB, *Ratio underweight* overweight/obesity

Similar results were observed when we separately analysed the children of immigrant parents and of Spanish parents separately, when we separately analysed the data from the children from the province of Cuenca and those from Ciudad Real separately in the 2007–2008 birth cohort (Additional file 2), and when the analyses, in both cohorts, were stratified by sex (Additional file 3).

The ORs for the association between three SES categories and weight status using logistic regression models, considering middle SES level as a reference category, were similar in both cohorts (Additional file 4).

## Discussion

The present study revealed that in both genders, in the cohort of children born in 1999–2000 the prevalence of underweight was lower and the prevalence of overweight/obesity was higher than in the cohort of 2007–2008. In addition, this study showed that the relationship between parental SES and children’s body composition has been substantially modified. While in the 1999–2000 cohort underweight was more frequent amongst children from high SES families and overweight/obesity was more frequent in children from low SES families, in the 2007–2008 children’s cohort the opposite was true.

The strengths of this study included that we have clearly described the procedures we followed to estimate and analyse our data. To our knowledge this is the only study that has examined the prevalence of underweight and overweight/obesity in Spanish children born before and after the 2008 financial crisis, and also examined the association between SES and weight status in these cohorts. We consider the data of this study very important from the point of view of public health as they suggest that Spanish children, the most vulnerable population group, are already suffering the consequences of the economic crisis; but also amongst other child populations where the social inequalities are growing which could be influencing their weight status distribution.

Our study had some limitations. First, the nature of the cross-sectional analysis of the baseline measurements data from two cluster randomised trials should be considered when interpreting the findings. Second, the age of the two cohorts was different at the measurement date, thus it is not possible to assume that changes in the prevalence of both overweight and obesity were not similar changes to those that have occurred in successive cohorts before and after the financial crisis; however, it is difficult to assume that stature percentiles at earlier ages do not continue to puberty and adolescence. Third, the distribution of SES changes over time, thus it is possible that changes in SES population distribution influence the statistical power in comparisons, therefore the absolute magnitude of differences should be taken into account. Fourth, the cohort effect prevents us from assuming that the same findings are valid for other cohorts before and after the financial crisis. Fifth, parental response rate for the SES questionnaire was considerably lower in the 1999–2000 cohort and this may have a bearing on the analysis of the results. Sixth, differences in children whose parents did respond to SES questions and those who did not could threaten the validity of the study, but Table 1 data indicated that the two subgroups were similar.

Childhood obesity continues to increase in some countries while in other countries it has apparently plateaued. A study in the US including data from 1999 to 2010 described an increasing trend in the prevalence of obesity amongst children and adolescents [18]. In Australia, studies published from 1996 to 2008 did not find substantial changes in childhood obesity prevalence [19]. A study in Switzerland reported that there was a decrease in obesity prevalence among children 6 through 13 years of age between 2002 and 2007 [20]. Our data providing information from two cohorts at different ages suggests that the children’s obesity epidemic could be starting to reverse, but new studies comparing children at the same age from different birth cohorts should be conducted in order to definitely clarify this concern, since the 2007–2008 figures might simply represent a cohort effect, and subsequent cohorts could return to previous figures.

International Obesity Task Force criteria (IOTF) were used in this analysis because their cut-offs for children are representative of the population of the whole world [17]. Predictions about the future prevalence of overweight in children from the industrialised countries are not consistent, since while some studies have warned about the need for effective interventions to reverse anticipated alarming trends [4, 21], other studies have emphasised the scarcity of data for early ages [22], and have even recently reported an unexpected stabilisation or decline in the prevalence of overweight in developed countries [23–25]. Our data showed that the prevalence of overweight/obesity in children from Castilla-La Mancha, Spain was lower in the 2007–2008 birth cohort than in 1999–2000, breaking down the upward trend that from 1992 to 2010 had been reported in the same population [1]. Several possible reasons have been argued to explain the stabilisation in childhood overweight/obesity rates, among them the cumulative effect of intervention programmes aimed at promoting healthy eating and physical activity, and also that the obesity epidemic has reached an equilibrium point between obesogenic environment and some degree of socially programmed resilience [23]. However, at least in the Mediterranean countries, we cannot rule out the influence of the financial crisis on the shocking increase of social inequality between 2007 and 2011 [26], such that 29.9 % of Spanish children and adolescents are estimated to live below the poverty line [27].

Since 1989 to 2005 the association between SES and adiposity in children from western developed countries was predominately inverse [28], thus it was widely accepted that the low-SES families from these countries were at greater risk of excess weight than the higher-SES families [29]. However, from the financial crisis, this view has been challenged. Our data showed that in the 1999-2000 children’s cohort adiposity was negatively associated with parental SES level, and in the 2007-2008 cohort the opposite was true. Several arguments might be outlined to explain this change in the relationship between parental SES and children adiposity, and among them the more reasonable might be related to the fact that subsidies for meals provided to children from low SES families in the schools have been removed during the last years [30], and it is conceivable that the most economically vulnerable families do not have sufficient resources to ensure minimum food for their children.

However, so far, the economic crisis impact on the health of the Spanish population is a debatable issue. A recent analysis reported the null impact of the economic crisis not only in premature mortality but also in self-reported health status [31]. However, another study predicted that, as the percentage of children below the poverty line has grown steadily from the onset of the financial crisis, the health consequences will soon appear [32] and, evidence suggests, might endure throughout a lifespan [33]. Our data showed that, as expected, the economic crisis impact has appeared in children of an early age, the most vulnerable population.

## Conclusions

Our data revealed that in both genders, underweight prevalence was lower in the cohort of children born in the wealthy years, and the prevalence of overweight and obesity was lower in the cohort of children born during the financial crisis. Also, our results showed that while in the 1999–2000 children’s cohort underweight was more frequent amongst children from high SES families and overweight/obesity was more frequent in children from low SES families, in the 2007–2008 children’s cohort the opposite was true. Therefore, our data should serve to alert Spanish and other countries’ welfare authorities about this issue, and might be useful to prioritise policies in order to minimise the impact of poverty on children’s development. Moreover, since no studies have compared the prevalence of underweight and excess weight in cohorts of children born before and during the 2008 economic recession, our data also highlight the convenience of monitoring growth indicators in children from cohorts born after the financial crisis.

## Additional files

Additional file 1:
**Differences in weight status by parental socio-economic status, controlling for age in the study, by birth cohort.** (DOC 34 kb) Additional file 2:
**Differences in frequencies of children in weight status categories by parental socio-economic status in birth cohort 2007–2008, controlling for age by parents nationalities and provinces.** (DOC 46 kb) Additional file 3:
**Differences in weight status by parental socio-economic status, controlling for age, by sex.** (DOC 46 kb) Additional file 4:
**Socioeconomic status** ^**1**^ **as predictor of underweight and overweight/obesity in logistic regression models controlling for age, by sex.** (DOC 35 kb)

## Abbreviations

SES: socioeconomic status
BMI: body mass index
IOTF: International Obesity Task Force
ANCOVA: analysis of covariance
OR: Odds ratio
