# Supplementary material for: Association between parental socioeconomic status with underweight and obesity in children from two Spanish birth cohorts: a changing relationship
Source: BMC Public Health. 2015 Dec 22;15:1276. doi: 10.1186/s12889-015-2569-5 (PMC4687138; doi:10.1186/s12889-015-2569-5)
Supplement: Additional file 1: — Differences in weight status by parental socio-economic status, controlling for age in the study, by birth cohort. (DOC 34 kb) [file 12889_2015_2569_MOESM1_ESM.doc]

**Additional file 1.** Differences in weight status by parental socio-economic status, controlling for age in the study, by birth cohort.

| Birth cohort 1999-2000 (n=544) Birth cohort 2007-2008 (n=1398)   |  | | --- | |  | | | | | | | | |  |
| --- | --- | --- | --- | --- | --- | --- | --- | --- | --- | --- |
| Parental socio-economic status (3 categories) | | | | | | | |  |
|  | Lower/Lower middle  n=112 | Middle  n=208 | Upper middle/Upper  n=224 |  | Lower/Lower middle  n=431 | Middle  n=627 | Upper middle/Upper  n=340 | |
|  |  |
| Underweight | 7.1  (1.9-12.3) | 7.7  (3.8-11.5) | 7.6  (3.9-11.3) |  | 21.6  (17.6-25.6) | 21.0  (17.8-24.3) | 18.2  (14.0-22.5) | |
| Normal weight | 51.8  (42.1-61.5) | 59.1  (52.2-66.0) | 58.5  (51.8-65.1) |  | 56.8  (52.0-61.6) | 59.0  (55.1-62.9) | 62.4  (57.0-67.6) | |
| Overweight | 25.9  (17.3-34.4) | 25.5  (19.3-31.6) | 26.3  (20.3-32.3) |  | 12.3  (9.1-15.5) | 11.3  (8.8-13.9) | 12.9  (9.2-16.6) | |
| Obesity | 15.2  (8.1-22.3) | 7.7  (3.8-11.5) | 7.6  (3.9-11.3) |  | 9.3  (6.4-12.1) | 8.6  (6.3-10.9) | 6.5  (3.7-9.2) | |
| Overweight/ obesity | 41.1  (31.5-50.6) | 33.2  (26.5-39.8) | 33.9  (27.5-40.3) |  | 21.6  (17.6-25.6) | 19.9  (16.7-23.1) | 19.4  (15.1-23.8) | |
