# Supplementary material for: Association between parental socioeconomic status with underweight and obesity in children from two Spanish birth cohorts: a changing relationship
Source: BMC Public Health. 2015 Dec 22;15:1276. doi: 10.1186/s12889-015-2569-5 (PMC4687138; doi:10.1186/s12889-015-2569-5)
Supplement: Additional file 2: — Differences in frequencies of children in weight status categories by parental socio-economic status in birth cohort 2007–2008, controlling for age by parents nationalities and provinces. (DOC 46 kb) [file 12889_2015_2569_MOESM2_ESM.doc]

|  | **Additional file 2.** Differences in frequencies of children in weight status categories by parental socio-economic status in birth cohort 2007-2008, controlling for age by parents nationalities and provinces. | | | | | | | | | | |
| --- | --- | --- | --- | --- | --- | --- | --- | --- | --- | --- | --- |
|  | Birth cohort 2007-2008 (n=1398) | | | | | | | | | |  |
|  | Parental socio-economic status (3 categories) | | | | | | | | | |  |
| Spanish (n=1178) | | | | | |  | | Immigrants (n=213) | | | |
|  | | Lower/Lower middle | Middle | Upper middle/ Upper |  | | Lower/Lower middle | | Middle | Upper middle/ Upper | |
|  | | n=308 | n=565 | n=305 |  | | n=122 | | n=60 | n=31 | |
| Underweight | | 21.4  (16.7-26.2) | 20.7  (17.3-24.1) | 18.0  (13.5-22.5) |  | | 22.1  (14.3-29.9) | | 25.0  (13.2-36.8) | 22.6  (6.2-38.9) | |
| Normal weight | | 57.1  (51.4-62.8) | 60.4  (56.2-64.5) | 62.3  (56.7-67.9) |  | | 55.7  (46.5-65.0) | | 48.3  (34.8-61.8) | 58.1  (39.1-77.0) | |
| Overweight | | 12.7  (8.8-16.5) | 11.0  (8.3-13.6) | 13.1  (9.2-17.1) |  | | 11.5  (5.4-17.5) | | 13.3  (3.9-22.8) | 12.9  (3.6-29.8) | |
| Obesity | | 8.8  (5.4-12.1) | 8.0  (5.6-10.3) | 6.6  (3.6-9.5) |  | | 10.7  (4.8-16.5) | | 13.3  (3.9-22.8) | 6.5  (0.8-21.4) | |
| Overweight/ obesity | | 21.5  (16.7-26.2) | 19.0  (15.6-22.2) | 19.7  (15.0-24.3) |  | | 22.2  (14.3-29.9) | | 26.6  (14.6-38.7) | 19.4  (7.4-37.5) | |
| Cuenca (n=509) | | | | | |  | | Ciudad Real (n=889) | | | |
|  | | Lower/Lower middle | Middle | Upper middle/ Upper |  | | Lower/Lower middle | | Middle | Upper middle/ Upper | |
|  | | n=168 | n=224 | n=117 |  | | n=263 | | n=403 | n=223 | |
| Underweight | | 25.0  (18.1-31.8) | 18.8  (13.4-24.1) | 13.7  (7.0-20.3) |  | | 19.4  (14.4-24.4) | | 22.3  (18.1-26.5) | 20.6  (15.1-26.2) | |
| Normal weight | | 56.5  (48.7-64.3) | 60.3  (53.6-66.9) | 63.2  (54.1-72.4) |  | | 57.0  (50.9-63.2) | | 58.3  (53.4-63.2) | 61.9  (55.3-68.5) | |
| Overweight | | 11.9  (6.7-17.1) | 9.4  (5.3-13.4) | 16.2  (9.1-23.3) |  | | 12.5  (8.3-16.7) | | 12.4  (9.1-15.7) | 11.2  (6.8-15.6) | |
| Obesity | | 6.5  (2.5-10.6) | 11.6  (7.2-16.0) | 6.8  (1.8-11.8) |  | | 11.0  (7.0-15.0) | | 6.9  (4.3-9.5) | 6.3  (2.9-9.7) | |
| Overweight/ obesity | | 18.4  (12.3-24.6) | 21.0  (15.4-26.5) | 23.0  (15.0-31.1) |  | | 23.5  (18.2-28.9) | | 19.3  (15.4-23.3) | 17.5  (12.3-22.7) | |
