# Supplementary material for: Association between parental socioeconomic status with underweight and obesity in children from two Spanish birth cohorts: a changing relationship
Source: BMC Public Health. 2015 Dec 22;15:1276. doi: 10.1186/s12889-015-2569-5 (PMC4687138; doi:10.1186/s12889-015-2569-5)
Supplement: Additional file 3: — Differences in weight status by parental socio-economic status, controlling for age, by sex. (DOC 46 kb) [file 12889_2015_2569_MOESM3_ESM.doc]

**Additional file 3.** Differences in weight status by parental socio-economic status, controlling for age, by sex.

| Boys | | | | | | | | | |  |
| --- | --- | --- | --- | --- | --- | --- | --- | --- | --- | --- |
| Birth cohort 1999-2000 (n=253) Birth cohort 2007-2008 (n=715) | | | | | | | | | |  |
|  | Lower/Lower middle  n=50 | Middle  n=105 | Upper middle/Upper  n=98 |  | Lower/Lower middle  n=232 | | Middle  n=308 | Upper middle/Upper  n=340 | | |
|  |  |
| Underweight | 8.0  (2.2-19.2) | 7.6  (2.1-13.2) | 8.2  (2.2-14.1) |  | 20.7  (15.3-26.1) | | 20.1  (15.5-24.8) | 17.7  (13.4-21.8) | | |
| Normal weight | 50.0  (35.1-64.8) | 60.0  (50.1-69.8) | 51.0  (40.6-61.4) |  | 59.5  (52.9-66.0) | | 60.1  (54.4-65.7) | 65.7  (60.4-70.8) | | |
| Overweight | 24.0  (11.2-36.8) | 24.8  (16.0-33.5) | 33.7  (23.8-43.5) |  | 11.6  (7.3-16.0) | | 10.7  (7.1-14.3) | 10.3  (6.9-13.7) | | |
| Obesity | 18.0  (8.1-22.3) | 7.6  (2.1-13.2) | 7.1  (1.5-12.7) |  | 8.2  (4.4-11.9) | | 9.1  (5.7-12.5) | 6.3  (3.5-8.9) | | |
| Overweight/ obesity | 42.0  (27.3-56.7) | 32.4  (22.9-41.8) | 40.1  (29.6-50.0) |  | 19.8  (14.5-25.2) | | 19.8  (15.2-24.4) | 16.6  (12.4-20.6) | | |
| Girls | | | | | | | | | |  |
| Birth cohort 1999-2000 (n=291) Birth cohort 2007-2008 (n=683) | | | | | | | | | |  |
|  | Lower/Lower middle  n=62 | Middle  n=103 | Upper middle/Upper  n=126 |  | Lower/Lower middle  n=199 | | Middle  n=319 | Upper middle/Upper  n=165 | | |
|  |  |
| Underweight | 6.5  (1.8-15.7) | 7.8  (2.1-13.4) | 7.1  (2.2-12.0) |  | 22.6  (16.5-28.7) | | 21.9  (17.2-26.6) | 18.8  (12.5-25.0) | | |
| Normal weight | 53.2  (40.0-66.4) | 58.3  (48.2-68.3) | 64.3  (55.5-73.0) |  | 53.8  (46.6-60.9) | | 58.0  (52.4-63.6) | 58.8  (51.0-66.6) | | |
| Overweight | 27.4  (15.5-39.3) | 26.2  (17.2-35.2) | 20.6  (13.2-28.1) |  | 13.1  (8.1-18.0) | | 11.9  (8.2-15.6) | 15.8  (10.0-21.6) | | |
| Obesity | 12.9  (3.7-22.0) | 7.8  (2.1-13.4) | 7.9  (2.8-13.0) |  | 10.6  (6.0-15.1) | | 8.2  (5.0-11.3) | 6.7  (2.5-10.8) | | |
| Overweight/ obesity | 40.3  (27.3-53.3) | 34.0  (24.3-43.6) | 28.5  (20.3-36.8) |  | 23.7  (17.5-29.8) | | 20.1  (15.5-24.6) | 22.5  (15.7-29.1) | | |
|  | | | |  | |  |  |  |  |  |
