# Supplementary material for: Association between parental socioeconomic status with underweight and obesity in children from two Spanish birth cohorts: a changing relationship
Source: BMC Public Health. 2015 Dec 22;15:1276. doi: 10.1186/s12889-015-2569-5 (PMC4687138; doi:10.1186/s12889-015-2569-5)
Supplement: Additional file 4: — Socioeconomic status 1 as predictor of underweight and overweight/obesity in logistic regression models controlling for age, by sex. (DOC 35 kb) [file 12889_2015_2569_MOESM4_ESM.doc]

**Additional file 4.** Socioeconomic status^1^ as predictor of underweight and overweight/obesity in logistic regression models controlling for age, by sex.

|  | Underweight | | Overweight/obesity | |
| --- | --- | --- | --- | --- |
|  | 1999-2000 | 2007-2008 | 1999-2000 | 2007-2008 |
|  | OR (95% CI) | OR (95% CI) | OR (95% CI) | OR (95% CI) |
| Boys |  |  |  |  |
| Lower/Lower middle | 0.79  (0.22-2.87) | 0.96  (0.62-1.49) | 0.64  (0.31-1.31) | 0.99  (0.63-1.54) |
| Upper middle/Upper | 1.25  (0.44-3.57) | 0.80  (0.49-1.31) | 1.49  (0.83-2.69) | 0.75  (0.45-1.24) |
|  |  |  |  |  |
| Girls |  |  |  |  |
| Lower/Lower middle | 1.02  (0.28-3.71) | 0.90  (0.58-1.41) | 0.73  (0.37-1.44) | 0.78  (0.50-1.22) |
| Upper middle/Upper | 0.83  (0.30-2.27) | 0.84  (0.51-1.37) | 0.76  (0.43-1.34) | 1.12  (0.69-1.79) |
|  |  |  |  |  |
| Total |  |  |  |  |
| Lower/Lower middle | 0.93  (0.37-2.30) | 0.94  (0.69-1.29) | 0.70  (0.43-1.14) | 0.88  (0.64-1.21) |
| Upper middle/Upper | 1.00  (0.48-2.06) | 0.82  (0.58-1.16) | 1.03  (0.69-1.56) | 0.92  (0.65-1.30) |

^1^Middle socioeconomic level as reference group.

Abbreviations: OR, Odds ratio; CI, confidence interval.
